# Comparative evaluation of 38% silver diamine fluoride on sealing ability of biodentine and MTA angelus as a perforation repair material: a confocal laser microscopic study

**DOI:** 10.1186/s12903-025-06656-1

**Published:** 2025-08-07

**Authors:** Saee Wazurkar, Aditya Patel, Manoj Chandak, Lalit Pawar, Sharvari Deshmukh

**Affiliations:** Department of Conservative Dentistry and Endodontics, Sharad Pawar Dental College and Hospital, Datta Meghe Institute of Higher Education and Research, Wardha, India

**Keywords:** Mineral trioxide aggregate, Biodentine, Perforation, Furcal perforation, Silver Diamine fluoride, Confocal laser microscopy

## Abstract

**Background:**

The objective of this research was to evaluate and compare the effect of 38% Silver Diamine Fluoride(SDF) on the sealing ability of Biodentine (BD) and MTA (Mineral Trioxide Aggregate) Angelus when utilized as a perforation repair material for human-extracted mandibular molars.

**Materials and methods:**

Forty human mandibular molars were extracted and divided into four groups (*N* = 10): Group 1 (BD), Group 2 (BD + SDF), Group 3 (MTA), and Group 4 (MTA + SDF). Standardized access cavities were prepared, and a 2 mm perforation was created at the pulpal floor. In Groups 2 and 4, SDF was applied for three minutes, followed by rinsing and air drying before placing BD or MTA. The restorations were then left to set for 48 h under humid conditions. Afterward, the specimens were immersed in a 0.6% rhodamine B dye solution for another 48 h. Each tooth was sectioned longitudinally, and dye penetration along the interface was examined under a confocal laser microscope at 40X magnification. Statistical analysis was performed using SPSS software, with one-way ANOVA and post hoc Tukey tests applied to determine significance (*p* < 0.05).

**Results:**

The intergroup analysis showed significant differences in dye penetration (*p* < 0.01). Group 3 (MTA) exhibited the least dye penetration followed by Group 2 (BD + SDF) and Group 4 (MTA + SDF). Group 1 (BD) demonstrated the highest dye penetration indicating the weakest sealing ability. The post hoc Tukey test confirmed that Group 1 had significantly higher microleakage than Groups 2, 3, and 4 (*p* < 0.001). While SDF significantly improved BD’s sealing ability (Group 2 vs. Group 1, *p* < 0.001), its effect on MTA was not statistically significant (Group 4 vs. Group 3, *p* = 0.530).

**Conclusion:**

MTA and BD, the primary materials used for perforation repair, face challenges related to solubility. Since SDF is nearly insoluble, it has the potential to form a protective barrier, enhancing marginal sealing and minimizing microleakage. However, there is currently no research examining how SDF influences the microleakage of MTA and BD in perforation repair. This in vitro study seeks to assess the impact of SDF on the sealing properties of these calcium silicate-based materials, providing valuable insights into improving perforation repair techniques.

## Introduction

In endodontic procedures, the infected pulp is removed, and gutta-percha is used to restore the root canal. Accuracy in diagnosis, access cavity preparation, biomechanical instrumentation, and obturation is crucial for successful endodontic treatment, as procedural errors can lead to complications. The prognosis is greatly impacted by endodontic iatrogenesis, which includes ledge formation, canal blockages, instrument separation, and iatrogenic perforations [1]. Furcal perforation, a serious complication of access cavity preparation, results in an iatrogenic communication between the periodontium and the root canal. It can result from caries, resorption, or procedural errors. The size, location, microbial contamination, sealing capacity of repair materials, and periodontal health of the perforation all affect the prognosis [1]. Perforations contribute to 9.62% of endodontic failures, with Seltzer et al. (1970) identifying them as the cause of 3.52% of failures. If untreated, they allow microbial invasion, leading to abscesses, fistulae, periodontal infections, and potential tooth loss. Effective management requires antimicrobial, biocompatible, and sealing repair materials [2]. Materials that have been used for furcal perforation repair include amalgam, glass ionomer cement (GIC), composites, Cavit, Super Ethoxy benzoic acid, and Intermediate Restorative Material (IRM); however, none have met al.l the ideal criteria. Mineral Trioxide Aggregate (MTA) was introduced by Arens and Torabinejad as a potentially superior alternative. MTA consists of dicalcium silicate, tricalcium aluminate, calcium oxide, and bismuth oxide. It sets into a colloidal gel, providing excellent sealing properties and biocompatibility [3].

MTA demonstrates favorable performance in moist environments and promotes healing by stimulating cementum formation. It resists marginal leakage by expanding slightly in moisture. Sluyk et al. (1998) reported MTA requires three days to achieve optimal sealing. Soundappan et al. (2014) demonstrated its superior sealing ability in comparison to Biodentine and IRM. Nevertheless, MTA presents certain limitations, such as a prolonged setting time, potential for discoloration, and challenges in handling [4–6].

Biodentine (BD) by Septodont (France) was developed as a fast-setting calcium silicate alternative. BD exhibits superior physical, biological, and mechanical properties, facilitating hard tissue regeneration with minimal associated inflammation. With a rapid setting time (9–12 min), BD is widely used for perforation repair. Aggarwal et al. (2013) found BD had a higher 24-hour push-out bond strength than MTA, and blood contamination affected MTA Plus more than BD [7–9].

Microleakage can compromise the success of endodontic treatment by increasing the risk of bacterial ingress. Calcium silicate-based materials form surface apatite crystals upon phosphate exposure, enhancing sealing. Caron G et al. (2014) found BD had more extraordinary sealing ability than MTA. Katge FA (2016) reported more dye penetration in MTA than BD. Shishir Singh (2015) found BD exhibited more excellent solubility than MTA, IRM, and GIC. Alazrag (2020) concluded that Theracal LC had the lowest solubility compared to MTA and BD. Both MTA and BD present advantages and disadvantages in the context of perforation repair. MTA remains the gold standard, while BD has shown promising results. MTA has a longer setting time and lacks antibacterial properties compared to BD but better promotes bone healing and reduces inflammation. Caron G. et al. (2014) noted BD has inferior radiopacity due to zirconium dioxide. BD exhibits strong mechanical properties but lower washout resistance than MTA [9–11].

Silver diamine fluoride (SDF) is a clear liquid that combines the remineralizing qualities of fluoride with the antibacterial qualities of silver, positioning it as a promising treatment for dental caries. Its ability to reduce certain cariogenic bacteria and its capacity to remineralize enamel and dentin are substantiated by numerous in vitro studies [12]. AgF solution was utilized in dentistry as early as the 1970s, according to Craig et al. Since the 1960s, the Central Pharmaceutical Council of the Japanese Ministry of Health and Welfare has approved SDF, a comparable compound, as a therapeutic agent for use in dental care. Numerous in vivo studies have been conducted to elucidate the precise mechanism of action of SDF; however, a complete understanding remains elusive. It is currently understood that the fluoride component reduces the solubility of the acid by products of bacterial metabolism and strengthens the tooth structure under acid attack, SDF may also disrupt the biofilm and kill the bacteria that cause the local environmental imbalance that demineralizes dental tissues [13]. Another investigation reported that the application of SDF led to the formation and deposition of less soluble or nearly insoluble compounds, including calcium fluoride, silver phosphate, and silver protein, on the surface of dentin [14]. A study by S. Osama (2024) investigated the impact of SDF on the microleakage of flowable resin composite and glass ionomer cement (GIC). The study concluded that SDF does not influence the microleakage of restorations bonded to carious dentin [15].

Solubility concerns exist with MTA and BD, which are commonly employed materials for perforation repair. Considering the near insolubility of SDF, it may function as a protective layer, improving marginal sealing and reducing microleakage. However, no studies have explored the effect of SDF on MTA and BD microleakage in perforation repair. This in vitro study aims to evaluate the impact of SDF on the sealing properties of these calcium silicate-based materials, offering new insights into enhancing perforation repair outcomes.

## Materials and methodology

This study was conducted within the Department of Conservative Dentistry and Endodontics, Sharad Pawar Dental College and Hospital (SPDCH), DMIHER, Sawangi, Wardha. This in vitro experimental study received approval from the Institutional Ethical Committee with Approval No.– DMIHER (DU)/IEC/2023/581. The study was conducted in line with Mohan D et al. (2021) study [16] employed SPSS software (SPSS 21–0 V, Claincalc version) to determine the sample size.

Sample size calculation, considering the anticipated effect of SDF on dye penetration in MTA and Biodentine groups, was performed with a power of 90% and a 95% confidence interval. It was found that the sample size per group was 10.

### Sample grouping

The specimen source was forty extracted mandibular molars procured from the Oral and Maxillofacial Surgery Department, SPDCH, DMIHER, Wardha. Each individual gave consent before the tooth was extracted.

Infection control protocol for the teeth obtained for this study The following suggestions and criteria by the “Occupational Safety and Health Administration (OSHA) and the Centres for Disease Control and Prevention (CDC)” were adhered.

### Inclusion and exclusion criteria

#### Inclusion criteria

- Permanent mandibular molars,
- Completely formed roots,
- Absence of caries and root canal filling.

#### Exclusion criteria

- root fractures,
- open apex,
- teeth with instances of internal or external resorption,
- teeth exhibiting cracks or fractures.

#### Materials and methods

**Fig. 1:**
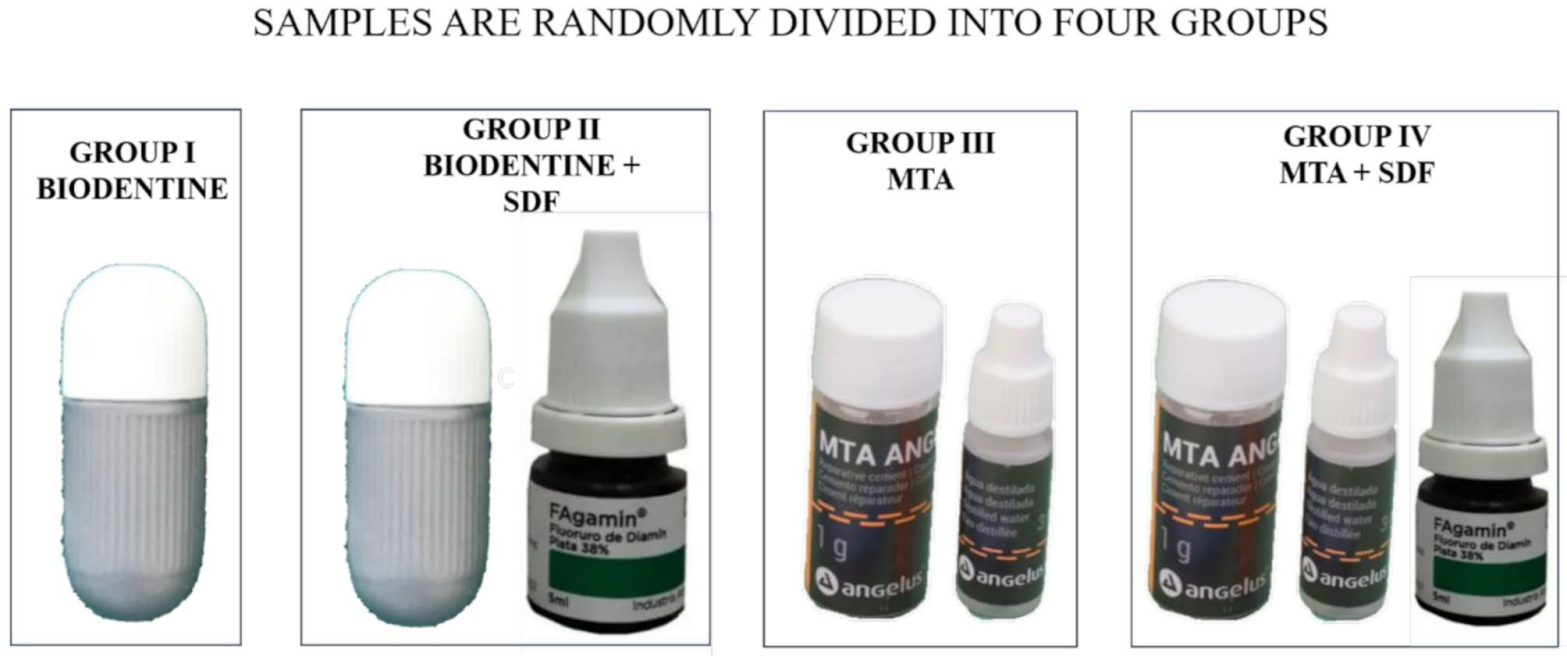
Sample size distribution

Forty freshly extracted human mandibular molars were utilized for this study, equally categorized into four groups, each comprising ten specimens (*N* = 10) Table 1. A standardized access cavity preparation was performed employing a BR-41 round bur (Mani, Japan), followed by the extension of the preparation using an EX-24 safe end bur (Mani, Japan). A perforation of 2 mm thickness was meticulously created at the center of the pulpal floor utilizing a round bur (Mani, Japan) in conjunction with a high-speed rotating handpiece to ensure consistency across samples. The specimens were organized into four distinct groups (G1-G4), with ten samples allocated to each group Fig. 1.

**Table 1:** Sample size distribution

Group I	Biodentine without application of 38% SDF	*N* = 10
Group II	Biodentine with the application of 38% SDF	*N* = 10
Group III	MTA Angelus without application of 38% SDF	*N* = 10
Group IV	MTA Angelus with the application of 38% SDF	*N* = 10

**Table 2:** List of materials used

Sr. No	Commercial name	Composition	Manufacturer
1	Biodentine	Powder- Tricalcium silicate (80.1%), calcium carbonate (14.9%), zirconium oxide (5%) Liquid- aqueous mixture of hydrosoluble polymer and calcium chloride	Septodont, France
2	FAgamin^®^	Contains 5 ml of 38% silver diamine fluoride solution at controlled pH	Tedequim SRL,– Argentina
3	Angelus MTA	Powder: Portland cement (75%), Bismuth Oxide (20%), and gypsum (5%). Portland cement is a mixture of Tricalcium silicate (CaO)3SiO2, Dicalcium silicate (CaO)2SiO2, Tricalcium aluminate (CaO)3 Al2O3, and Tetracalcium aluminoferrite (CaO)4Al2O3Fe2O3 Liquid: distilled water	Londrina, Brazil
4	Rhodamine dye	Rhodamine B base (a powder), dissolved in ethanol	Shivay Enterprise, Surat India

### Repair of the perforations (Table 2)

#### Group 1 BD (G1)

**Fig. 2:**
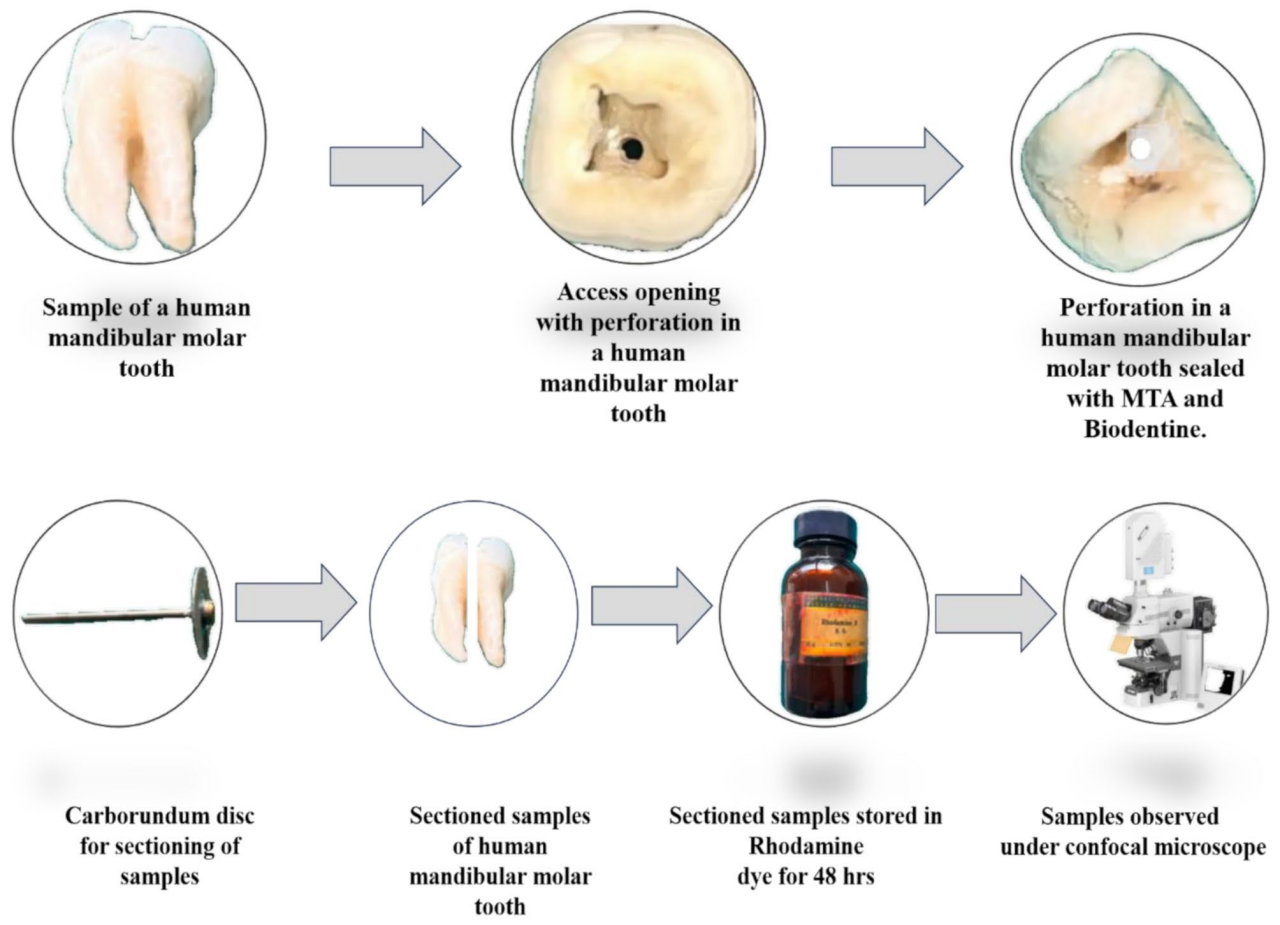
Stepwise procedures of the study performed

BD was mixed according to the manufacturer’s instructions until ideal consistency was achieved. The powder was mixed mechanically with liquid placed in the perforations with a spatula slightly condensed with Amalgam pluggers and allowed to set for 12 min. A saline–dipped damp cotton was placed on the restoration for 48 h (Fig. 2).

#### Group 2 BD + SDF (G2)

**Fig. 3:**
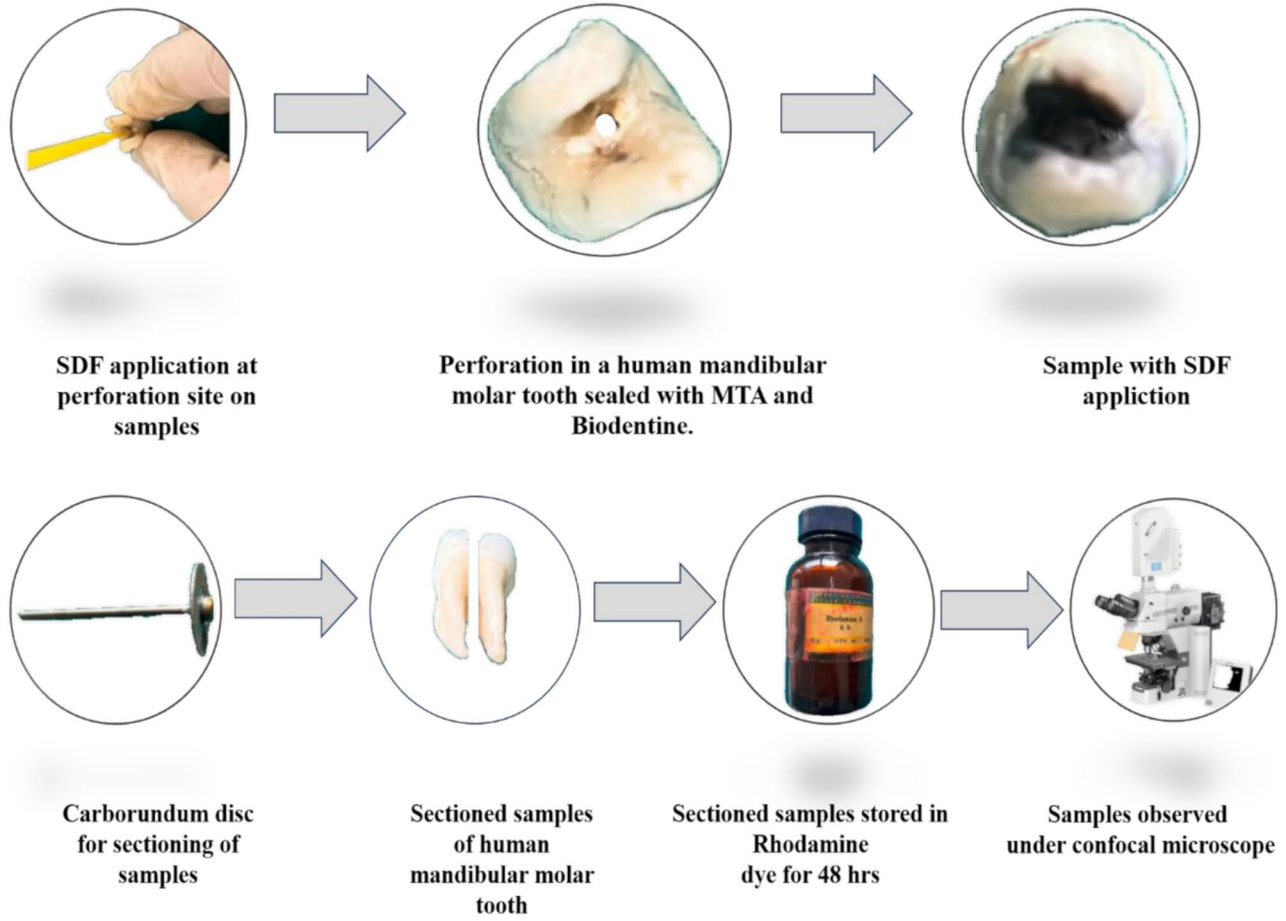
Stepwise procedures of the study performed

SDF was applied on the walls of the perforation site, using a micro-tip applicator, and kept for 3 min, it was further rinsed for 30 s and air-dried for 5 s, BD was mixed according to the manufacturer’s instructions until ideal consistency was achieved, the powder was mixed mechanically with liquid placed in the perforations with a spatula slightly condensed with Amalgam pluggers, and allowed to set for 12 min. A saline–dipped damp cotton was placed on the restoration for 48 h Fig. 3.

#### Group 3 MTA (G3)

MTA Angelus was mixed according to the manufacturer’s instructions to achieve a homogenous mix, placed using the MAP system (Master Apical Placement, Produits Dentaires, Switzerland), and condensed using a hand plugger into the perforation defect. Similar steps were performed as in Fig. 2. A saline-dipped damp cotton ball was placed over MTA for 48 h.

#### Group 4 MTA + SDF (G4)

SDF was applied on the walls of the perforation site, using a micro-tip applicator, and kept for 3 min, it was further rinsed for 30 s, and air-dried for 5 s, over which MTA was placed using the MAP system (Master Apical Placement, Produits Dentaires, Switzerland), and was condensed using a hand plugger into the perforation defect. A saline-dipped damp cotton ball was placed over MTA for 48 h Fig. 3.

In the subsequent 48 h, the dental specimens underwent treatment involving two applications of clear nail varnish, ensuring that a margin of 1–2 mm around the perforation site remained uncoated. Following this, the specimens were preserved in a controlled environment with 100% humidity and a temperature of 37 °C for an additional 48 h. Subsequently, the specimens were immersed in a 0.6% rhodamine B dye solution for a period of 48 h. Each tooth was longitudinally sectioned in a buccolingual orientation at the perforation site, utilizing a 0.3 mm thick diamond disc under water cooling to ensure precision. The maximum apical extent of dye penetration along the material-tooth interface was measured between the tooth structure and the repair material was measured under a confocal microscope at 40X magnification [17].

### Statistical analysis

Once the data collection was complete, statistical analysis was conducted using SPSS version 21. Using the mean, standard deviation, frequency, and percentage, the descriptive statistics were presented. The data distribution’s normality was assessed using the Shapiro-Wilk test results, which confirmed that it followed a normal distribution. To ascertain whether the means of the various groups differed significantly, a one-way ANOVA test and post hoc Tukey test were used. The significance level was set at *p* < 0.05.

## Results

### Findings

This study evaluated the effect of 38% Silver Diamine Fluoride on the sealing ability of Biodentine and MTA Angelus when used as a perforation repair material on human-extracted mandibular molars. The amount of microleakage was measured using the dye penetration method in (mm) using confocal laser microscopy.

**Fig. 4:**
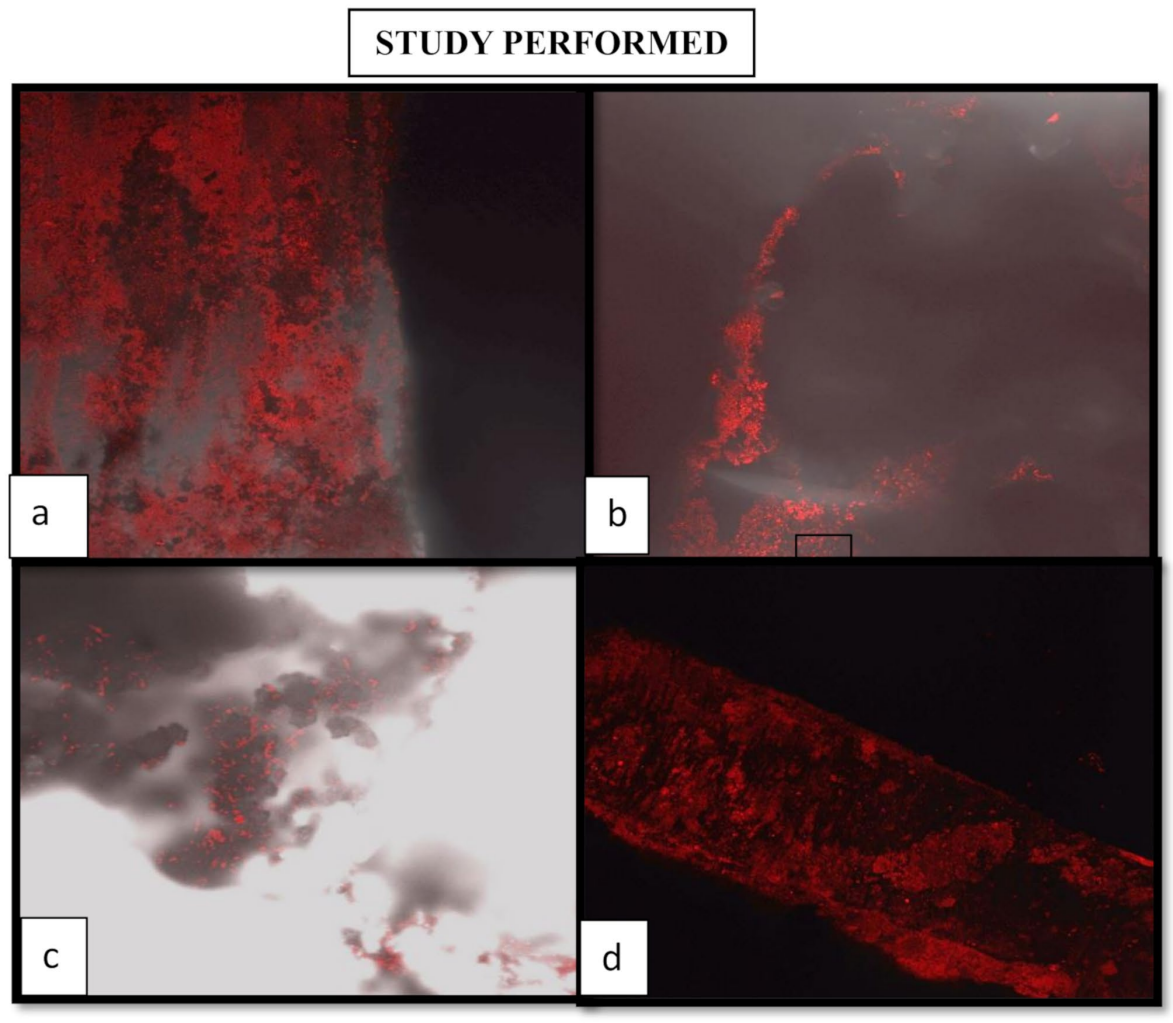
Confocal Laser Microscopic image( Fig 4). (**a**) The red fluorescence indicates dye penetration around the margins of the material, which suggests microleakage. The red fluorescence is more intense with increased dye penetration, indicating poorer sealing ability. Figure (**b**) shows moderate microleakage. This means it has some sealing ability, but not optimal sealing ability. It is better than (**a**) and shows less fluorescence; however, it still demonstrates leakage. Figure 4 (**c**) shows indications of some blurred fluorescence and scattered areas; this suggests surface interaction with the material rather than deep penetration, which suggests some degree of sealing ability and possibly a better sealing binding ability compared to figures (**a**) and (**b**). This may not produce complete sealing but may correspond to a sealing ability that is superior compared to figures (**a**) and (**b**). The scattered fluorescence areas may indicate slight microleakage, but those areas appear more isolated in the specimen compared to Figures (**a**) and (**b**). Figure 4 (**d**): The fluorescence appears to be far less deep and has a more uniform appearance; the minor penetration may indicate a more uniform adaptation of the material and a smaller number of gaps in the material. Again, fluorescence spots could indicate microleakage. (**a**) shows confocal laser microscopic image at 40X magnification of (Group 1). (**b**) shows confocal laser microscopic image at 40X magnification of (Group 2). (**c**) shows confocal laser microscopic image at 40X magnification of (Group 3). (**d**) shows confocal laser microscopic image at 40X magnification of (Group 4)

As shown in Table 3; the intergroup analysis of dye penetration in mm, the mean values for Group 1, Group 2, Group 3, and Group 4 were respectively (**0.147 ± 0.057 mm)**, **(0.053 ± 0.044 mm)**, **(0.035 ± 0.036 mm)**, **and (0.063 ± 0.043 mm)**. These values indicated a statistically significant difference, as the *p*-value was less than 0.01. Group 3 exhibited the least dye penetration, followed by Group 2 and Group 4, while Group 1 demonstrated the highest dye penetration among all groups.

**Table 3:** Intergroup comparison of dye penetration in mm between group 1, group 2, group 3, and group 4 using one-way ANOVA

Groups	*N*	Mean	Std. Deviation	Minimum	Maximum	F-test	*P*-value
Group 1	10	0.147	0.057	0.0093	0.2349	11.815	< 0.01
Group 2	10	0.053	0.004	0.0104	0.1358		
Group 3	10	0.035	0.036	0.0061	0.1358		
Group 4	10	0.063	0.043	0.0044	0.1358		

**Table 4:** Mean difference of dye penetration between group 1, group 2, group 3, and group 4 using post hoc Tukey HSD test

Group	Compared to	Mean Difference	SE	df	t	*p*-value	Level of Significance
Group 1	Group 2	0.094	0.020	36	4.612	< 0.001	Highly Significant
	Group 3	0.112	0.020	36	5.469	< 0.001	Highly Significant
	Group 4	0.084	0.020	36	4.106	0.001	Highly Significant
Group 2	Group 3	0.018	0.020	36	0.857	0.827	Not Significant
	Group 4	-0.010	0.020	36	-0.505	0.957	Not Significant
Group 3	Group 4	-0.028	0.020	36	-1.363	0.530	Not Significant

The mean difference is significant at the (*p* < 0.01)

The post hoc comparative assessment of dye penetration revealed significant differences among the groups. The dye penetration was significantly higher in G1 than in G2 (mean difference 0.094; *p* < 0.001), G3 (mean difference 0.112; *p* < 0.001), and G4 (mean difference 0.084; *p* = 0.001). According to these results, G1 had the worst sealing ability, whereas the other groups were better at reducing dye penetration. G2 and G3 (mean difference = 0.018, *p* = 0.827) and Group 2 and Group 4 (mean difference = -0.010, *p* = 0.957) did not differ statistically likewise, there was no significant difference between Groups 3 and 4 (mean difference 0.028, *p* = 0.530)

## Discussion

The preservation of natural teeth is paramount for both functional and aesthetic purposes, and endodontic therapy is instrumental in achieving this objective [18]. However, procedural complications such as furcation perforation, which creates an opening into the periodontal ligament space, and accidental pulp chamber floor perforation can present significant challenges during treatment [19]. These perforations can induce an inflammatory response, potentially leading to periodontal damage, granulomatous tissue formation, epithelial proliferation, and periodontal pocket development [20]. Studies indicate that root perforations occur in approximately 2–12% of endodontic cases. Although this percentage may seem relatively low, such perforations can significantly affect treatment outcomes, as they are strong predictors of the need for retreatment [21]. Additionally, compared to teeth without perforations, the healing rate of teeth with perforations was found to be significantly lower by 31%, highlighting the significance of preventing perforations during initial procedures [21].

A critical determinant for the successful repair of furcation perforations is the establishment of a three-dimensional hermetic seal. The success of this seal relies on a number of factors such as the volume stability of the material, its adhesive properties, solubility, and marginal adaptation. Fluid leakage and gap formation at the interface between the repair material and dentin serve as indicators of a material’s sealing ability [22]. Microleakage is one of the primary causes of restoration failure, postoperative sensitivity, and even tooth loss. Microleakage refers to the procedure through which bacteria, fluids, and other materials pass between the cavity walls and the restorative material. A variety of reasons are accountable for this procedure, including thermal expansion differences between tooth structure and the material, polymerization shrinkage, surface deterioration with time, and improper placement procedures. Since some of these issues are not always detectable by routine clinical examination, they pose an added challenge in the attainment of long-term treatment success [23].

Since its introduction in the 1990s, Mineral Trioxide Aggregate (MTA) has significantly transformed the technique of endodontic treatment, particularly where there is a requirement for a dry field such as in the treatment of the pulp, perforation repair, root-end fillings, and apexification. MTA is widely recognized for its exceptional sealing capacity, antibacterial properties, and biocompatibility. Furthermore, its hydrophilic nature enhances its performance in moist environments. A study conducted by Kumari et al. (2018) demonstrated that the solubility of MTA was lower compared to that of Biodentine (BD) as seen after 30 and 60 days. This lowered solubility is primarily attributed to the constituent bismuth oxide, a virtually insoluble compound. Their research also demonstrated that MTA allowed for the least amount of dye penetration, and therefore, this reflected improved sealing capacity [24, 25].

Conversely, BD, a calcium silicate-based material, exhibits higher solubility than MTA due to increased ion release. While BD has some advantages—e.g., shorter setting time and improved handling properties—it has poorer bond strength to dentine. BD is often promoted as a dentine substitute due to comparable mechanical properties to natural dentine. Perforation repair material solubility is an important consideration because it has a direct impact on sealing ability, biocompatibility, and adaptation to the environment. Studies have established BD to be more soluble than MTA, though marginal adaptation is comparable. Still, differences are not statistically significant. Das et al. (2022) compared microleakage of furcation perforations of extracted molars treated with MTA, BD, and Endosequence using scanning electron microscopy (SEM) under 2000x magnification. The findings showed that BD had higher sealing ability than MTA and Endosequence, possibly because it has a lower water-powder ratio, shorter setting time, and smaller particle size, resulting in a more uniform final structure (22,26).

Silver diamine fluoride (SDF) has gained popularity over the last few years in dentistry because it is antimicrobial and will deposit minerals in treated dentin to increase mineral content. SDF, upon application, forms a highly mineralized layer that is approximately 150 microns in thickness and more resistant to demineralization than healthy dentin [26]. SDF has been found to exert an effective antimicrobial effect on cariogenic biofilms, particularly those produced by Streptococcus mutans and Lactobacillus acidophilus [27]. SDF is also seen to protect the collagen of dentin from degradation. Upon interaction with dentin, SDF forms silver-protein complexes and an adherent layer of minerals, mainly in the form of silver phosphate and silver chloride. The layer is highly resistant to dissolution, which is an element that makes dentin strong and provides a long-lasting antimicrobial effect. According to a study conducted by Uzel et al. (2013), it was discovered that SDF application led to the formation of compounds such as calcium fluoride and silver phosphate that acted to inhibit the loss of calcium and phosphorus in carious lesions [19].

To date, no studies have specifically examined the impact of SDF on the microleakage properties of MTA and BD, presenting a notable gap in the literature. Addressing this gap, the present study employed a dye penetration method to evaluate the sealing ability of these materials. According to Camps and Pashley (2000), dye penetration analysis provides results comparable to the fluid filtration technique, as both methods quantify liquid movement along the material-tooth interface. This study used Rhodamine B dye due to its reliability in assessing microleakage through linear measurements [28]. Using a dye is considered one of the simplest and most cost-effective approaches for detecting microleakage. Rhodamine B dye was selected in this study due to its ability to enable quantitative assessment of dye penetration through linear measurements. Confocal laser scanning microscopy (CLSM) was selected as the primary analytical tool, as it offers a non-destructive means of evaluating the adaptation of dental materials to dentin. Unlike SEM, CLSM does not require sample dehydration, thereby minimizing the risk of shrinkage artifacts and improving the accuracy of assessments [29].

Other methods, such as bacterial leakage models, are considered more clinically relevant but come with significant drawbacks — they tend to be complicated, lengthy, difficult to reproduce, and are heavily influenced by variations in bacterial growth [30]. Fluid filtration techniques, although offering quantitative data, mainly measure permeability and do not fully evaluate the structural integrity of the material-tooth interface. Since the objective of this study was to assess and compare the sealing ability of different groups under consistent, controlled conditions, the dye penetration method combined with CLSM was selected as it offers a reliable, sensitive, and practical approach within the limitations of an in vitro setting.Mandibular molars were chosen for this study due to their anatomical complexity and higher susceptibility to furcal perforation. The relatively thin pulpal chamber floor near the furcation area increases the risk of perforation during endodontic procedures. The complex root anatomy of mandibular molars, including multiple roots and canals, increases the risk of procedural errors [31]. Moreover, the presence of multiple roots and intricate canal morphology further contributes to the likelihood of procedural errors. This study reported that the application of silver diamine fluoride (SDF) significantly decreased dye penetration in both Biodentine (BD) and mineral trioxide aggregate (MTA) groups. In one-way ANOVA, there was a significant difference in microleakage between the four groups (*p* < 0.001). Our post hoc Tukey analysis indicated BD had the most leakage and MTA had the least. (Table 4.) This supports the claim found in the literature that MTA generally provides a better long-term seal due to its hydrophilic nature and formation of a good interface with dentin, thereby minimizing marginal leakage [32].

In Group 2, Biodentine showed improved sealing when pretreated with silver diamine fluoride (SDF). This improvement is likely due to mineral deposits like calcium fluoride and silver phosphate forming within the dentinal tubules, which reduce permeability and strengthen the dentin barrier (Uzel et al., 2013 [33]; Osama et al., 2024 [15]. SDF’s antibacterial and collagen-stabilizing effects also help maintain the dentin-material interface, especially in areas prone to leakage, such as furcation perforations Mei et al., 2013 [13]; Hassanen et al., 2022 [26]. Group 1, using Biodentine without SDF, had the most dye penetration, likely due to its sensitivity to moisture and higher solubility Singh et al., [21]. 2015; Alazrag et al., 2020 [22]. While SDF improved Biodentine’s performance, it had little effect on MTA, which already showed strong sealing ability (*p* = 0.530).

In this study, sealing ability was evaluated after 48 h of setting and dye immersion to reflect the material’s early behavior. However, we recognize that this short period does not account for long-term stability, material degradation, or solubility changes in the oral environment. Research by Grech et al. 2013 [32], Kaup et al. 2015 [18], and Alazrag et al. 2020 ( 22) indicates that calcium silicate-based materials continue to mature and may behave differently over extended periods. Although MTA was found to have a better sealing ability than Biodentine in this study, other research has produced different results. For example, under SEM analysis, Das et al. 2022 [34] and Katge et al. 2016 [35] found less microleakage with Biodentine. Aggarwal et al. [8] 2013 and Caron et al. 2014 [9] also emphasised the clinical sealing and favourable early bond strength of Biodentine. Differences in methodology, setting time, particle size, and evaluation methods could be the cause of these disparities. Consequently, additional research employing long-term clinical models is necessary to validate these results.

## Conclusion

Based on the findings of this study, MTA remains a preferred material for perforation repair due to its superior sealing ability, hydrophilic properties, and capacity for interfacial layer formation. While SDF pretreatment resulted in a marginal improvement in MTA’s performance, it significantly enhanced the sealing properties of BD. SDF appears to enhance the performance of materials with intrinsically weaker sealing capabilities by reducing their solubility and forming a protective barrier. The results of this in vitro study suggest that BD may require pretreatment with SDF to achieve sealing properties comparable to MTA. Although MTA demonstrated favorable sealing ability independently, pretreatment with SDF presents a potential strategy to enhance the performance of materials with lower intrinsic sealing effectiveness, such as BD. The long-term clinical effects of these materials and methods, especially in different clinical settings, should be investigated further.

## Data Availability

The data that support the findings of this study are available from the corresponding author upon reasonable request.

## Declarations

### Clinical significance

Application of SDF as a layer of varnish on perforation defect decreases the solubility of calcium silicate-based material like MTA and BD and enhances its sealing efficacy.

## Abbreviations

MTA: Mineral Trioxide Aggregate
SDF: Silver Diamine Fluoride
BD: Biodentine
CLSM: Confocal Laser Microscopy
SEM: Scanning electron microscopy
GIC: Glass Ionomer Cement
IRM: Intermediate Restorative Material
